# Editorial: Community series in the immune escape mechanism and novel immunotherapeutic strategies of leukemia, volume II

**DOI:** 10.3389/fimmu.2024.1426570

**Published:** 2024-05-10

**Authors:** Yixiang Han, Qianping Li, Yicheng Tan, Kang Yu, Shenghui Zhang

**Affiliations:** ^1^ Central Laboratory, The First Affiliated Hospital of Wenzhou Medical University, Wenzhou, Zhejiang, China; ^2^ Department of Hematology, The First Affiliated Hospital of Wenzhou Medical University, Wenzhou, Zhejiang, China; ^3^ Institute of Hematology, Wenzhou Medical University, Wenzhou, Zhejiang, China; ^4^ Wenzhou Key Laboratory of Hematology, Wenzhou, Zhejiang, China; ^5^ Laboratory Animal Center, The First Affiliated Hospital of Wenzhou Medical University, Wenzhou, Zhejiang, China

**Keywords:** leukemia, immune escape, immunotherapeutic strategies, chimeric antigen receptor T-cell therapy, cytokine-release syndrome

Leukemia is a heterogenous group of hematologic malignancies characterized by not only the excess production of immature or abnormal leukocytes but also an altered immune response (1). The dysfunction and reprogramming of the immune system play critical roles in leukemia initiation and progression (2). Immunotherapy aims to reactivate antitumor immune cells and overcome the immune escape mechanisms of leukemia in the bone marrow microenvironment. Represented by hematopoietic stem cell transplantation (3), chimeric antigen receptor (CAR) T-cell therapy (4), and T cell receptor (TCR) T-cell therapy (5), immunotherapy for leukemia has achieved tremendous success in clinical practice. However, the overall prognosis of leukemia immunotherapy still needs improvement, which warrants the discovery of biomarkers as well as the development of novel therapeutic designs.

In this topic, Hino et al. summarized the role of the thymus in generating a diverse T lymphocyte repertoire and how age-related thymic involution and diseases such as acute myeloid leukemia (AML) affect T cell-mediated anti-tumor immunity. Patients with AML have impaired immune surveillance including reduced output of naïve T cells (6), restricted TCR repertoire (7), and increased frequency of regulatory T cells (8). Therefore, there is a potential correlation between thymus dysfunction and T-lymphocyte impairment with the ontogeny of AML. Many strategies for rejuvenating and boosting thymus function, such as administration of interleukin-7 and keratinocyte growth factor, have shown encouraging results in clinical trials for AML and other hematological malignancies, suggesting that understanding thymic involution and dysfunction in hematological malignancies is crucial to developing successful immunotherapies. Li et al. constructed a novel CD4^+^ T cell vaccine (CD4^+^ T_LEX-CD8086_) by combining CD4^+^ T cells with costimulatory molecules gene-modified leukemia cell-derived exosomes (LEXs), which harbors upregulated CD80 and CD86 to enhance immunogenicity. Through the acquisition of costimulatory molecules, these CD4^+^ T_LEX-CD8086_ cells can act as antigen presenting cells and are capable of directly stimulating the leukemic cell antigen-specific CD8^+^ cytotoxic T lymphocyte (CTL) response. Immunization of CD4^+^ T_LEX-CD8086_ cells can significantly delay the tumor growth and prolong the survival of tumor-bearing mice in both protective and therapeutic models. Therefore, the anti-leukemia vaccine may have promising potential for leukemia immunotherapy. Singh et al. found a strong negative correlation between the childhood leukemia incidence (LI) in populations aged 0-4-years with >90% childhood Bacille Calmette-Guerin (BCG) vaccination coverage and prevailing tuberculin immunoreactivity by analyzing the early childhood LI for 2020 in European Region countries. They hypothesize that early childhood BCG vaccination “priming” and subsequent “trained immunity” reinforcement by “natural” stimulating from Mycobacterium spp. exposure exert a preventive and protective role in childhood LI. Exploratory studies, preferably conducted in high-burden countries and controlling for the trained-immune correlations and other potential confounding factors, will be necessary to clarify the role of BCG vaccination and early immune training in childhood LI and help to end the current dispute. Luan et al. reported a case with ALL who developed local cytokine-release syndrome (L-CRS) following CAR-T therapy. While L-CRS is typically observed in compartmental tumors, this case represents the first reported instance of L-CRS occurring in systemic malignancies. The authors propose a possible model to explain the occurrence and development of L-CRS in systemic malignancies, suggesting that L-CRS may occur as a result of the redistribution of CAR-T cells and cytokines after the elimination of tumor cells. This study serves as a reminder that patients with systemic malignancies can also suffer from L-CRS, necessitating meticulous evaluation for potential emergent interventions such as vigilant monitoring, aggressive supportive or immunosuppressive agents.

In recent years, tremendous approaches targeting the adaptive immune system have achieved great success in the treatment of hematological malignancies (9). Due to the innate immune system being the first line of defense against the external environment, its targeted therapies may offer additional hope for the treatment of hematological malignancies. CD47 as a cell surface ligand is overexpressed in a variety of malignant tumors and binds to signal-regulating protein alpha (SIRPa) on macrophages to promote tumor cells to escape phagocytosis. Blocking CD47-SIRPa axis can increase phagocytosis of macrophages and exert anti-tumor effect. Inhibitors targeting the CD47-SIRPa axis are being developed worldwide and undergoing preclinical and clinical studies. Xu et al. focused on the opportunities and challenges of anti-CD47 antibodies in hematologic malignancies. The combinations of anti-CD47 antibodies with other drugs have shown encouraging treatment response rate in patients with blood tumors, but side effects such as anemia also occur. Furthermore, bispecific antibodies and SIRPa/Fc fusion proteins appear to solve these difficulties and efficiently balance the efficacy and safety of the treatment.

Cao et al. reported two cases of B-cell acute lymphoblastic leukemia (B-ALL) patients with central nervous system (CNS) involvement who received blinatumomab, a CD3/CD19 bispecific antibody. Case 1 was diagnosed with chronic myeloid leukemia and developed B-ALL with CNS involvement during treatment with dasatinib, and Case 2 was diagnosed with B-ALL and experienced early hematologic relapse and cerebral parenchyma involvement. Both patients achieved complete remission in the bone marrow and CNS after treatment with blinatumomab, suggesting that blinatumomab may be a potential promising treatment option for B-ALL patients with CNS leukemia. Xu et al. comprehensively explored the clinical benefits and safety of gemtuzumab ozogamicin (GO), an anti-CD33 humanized antibody in the treatment of AML. They summarized that GO tended to improve complete remission rates, followed by significantly improved survival outcomes including overall survival, event-free survival, relapse-free survival, and cumulative incidence of relapse. Subgroup analysis indicated that GO benefits were evident in patients with favorable- and intermediate-risk karyotypes, NPM1 mutations, and wild-type FLT3-ITD. Furthermore, administration of GO was also associated with reduced relapse rates and improved survival in certain patient subgroups, such as those aged <70 years, with *de novo* AML, and with CD33^+^. Incorporating GO into established induction treatment strategies and a lower (<6 mg/m^2^) dose of GO prolonged survival, while administration of GO increased risk of earth death at a higher dose (≥6 mg/m^2^) and enhanced hepatic-related adverse effects. Further head-to-head randomized controlled trials need be performed to validate the therapeutic benefits and safety of GO in AML. Wu et al. systematically summarized the role of CD44 in leukemia development, which can promote the proliferation, migration, metastasis, chemoresistance and immune evasion of leukemic cells. CD44 and its variants as biomarkers are associated with poor prognosis, relapse or drug resistance in leukemia patients. Some CD44 subtypes, especially CD44v6 and CD44v3 tightly interact with the extracellular matrix, such as hyaluronic acid (HA) and osteopontin, to activate Nanog to enhance the expression of drug-resistance genes. Given the powerful tumor promoting ability of CD44 and its high affinity with HA, a number of CD44-related interventions, including anti-CD44 monoclonal antibodies, specific small molecular drugs, and HA-directed targeting of cancer cells, have been applied in the clinical treatment of leukemia. These strategies have shown encouraging results in preclinical and clinical trials. Certainly, targeting CD44 inevitably causes some side effects due to its expression on certain immune cells.

Overall, the combination of substantial research on leukemia immunology and advanced technology for manipulating immune cells will enlighten the future development of leukemia immunotherapy. The advancement of immunotherapy for leukemia calls for more integrated clinical and basic research programs to comprehensively analyze unmet clinical needs and subsequently guide research directions.

## Author contributions

YH: Writing – original draft. QL: Writing – original draft. YT: Writing – original draft. KY: Writing – review & editing. SZ: Conceptualization, Writing – review & editing.
